# What’s in a Name? Utilization of the Innominate Vein for Pacemaker Lead Placement in the Setting of Persistent Left Superior Vena Cava

**DOI:** 10.7759/cureus.1057

**Published:** 2017-02-26

**Authors:** Mohammad-Ali Jazayeri, Rehan Karim

**Affiliations:** 1 Cardiovascular Research Institute, University of Kansas Hospital & Medical Center; 2 Division of Cardiology, Hennepin County Medical Center, University of Minnesota Medical School

**Keywords:** left superior vena cava, plsvc, congenital anomaly, permanent pacemaker, lead placement, innominate vein, electrophysiology, cardiology, cardiac implantable electronic device, fluoroscopy

## Abstract

Persistent left superior vena cava (PLSVC) represents the most common thoracic venous anomaly and is an important clinical entity for cardiologists and electrophysiologists, among others. In approximately 30% of cases, a bridging innominate vein connects the left superior vena cava to the right. The present report highlights the value of defining the venous anatomy with a case of dual-chamber pacemaker implantation in the PLSVC with the right ventricular lead placed via the innominate vein. Pertinent considerations for device implantation in the setting of this anomaly are discussed and relevant venography reviewed.

## Introduction

Persistent left superior vena cava (PLSVC) represents the most common intrathoracic venous anomaly with an estimated prevalence of 0.2% in the general population and those undergoing pacemaker or implantable cardioverter defibrillator (ICD) implantation [1-2]. Moreover, among patients with other congenital cardiac anomalies, this percentage has been observed to be significantly higher in large pathologic studies [1]. Though typically PLSVC is clinically silent and incidentally detected in the absence of other cardiac defects, it can be hemodynamically significant when in direct communication with the left atrium and poses technical challenges with respect to electrophysiological procedures, cardiac surgery, and central venous cannulation.

Informed consent was obtained from the patient for this study.

## Case presentation

A 55-year-old male with no significant past medical history presented to the emergency department with symptoms of acute-onset dizziness, nausea resulting in emesis, and pre-syncope. The electrocardiogram (ECG) demonstrated a high degree atrioventricular (AV) block with pauses of up to 10 seconds in duration. Following negative evaluation for reversible causes for his condition, he underwent dual-chamber pacemaker implantation.

During the procedure, venous access was obtained by way of the left subclavian vein, which required some negotiation during the advancement of the guidewire. Resistance was noted at the time of sheath advancement, prompting re-evaluation with pullback that confirmed intravenous positioning with normal flushing of the sheath. A venogram was obtained, demonstrating an atretic subclavian-innominate vein system draining into a patent right superior vena cava (Video 1).

**Video 1:** Atretic right subclavian-innominate vein system Venogram demonstrating an atretic subclavian-innominate vein system draining into a patent right superior vena cava.

Further retraction of the sheath with repeat venography demonstrated a large PLSVC draining into the right atrium (RA) via an enlarged coronary sinus (Video 2). The standard sheath accessing the atretic bridging innominate vein was changed to a Wholey wire for increased maneuverability, and the vessel was traversed with a 7-French braided coronary sinus sheath. Through this sheath, a right ventricular (RV) pacing lead was advanced and successfully implanted into the RV apical septum. A 7-French short sheath positioned in the coronary sinus via the PLSVC was used to place the right atrial (RA) lead in its posterolateral wall (Video 3). Final pacing thresholds were 0.9 V at 0.5 ms and 0.5 V at 0.5 ms for the RA and RV leads, respectively.

**Video 2:** Large PLSVC draining into the right atrium Venogram demonstrating a large PLSVC draining into the right atrium via an enlarged coronary sinus.

**Video 3:** Final pacemaker lead position Venogram showing final dual-chamber pacemaker lead position in the setting of PLSVC.

**Figure 1:**
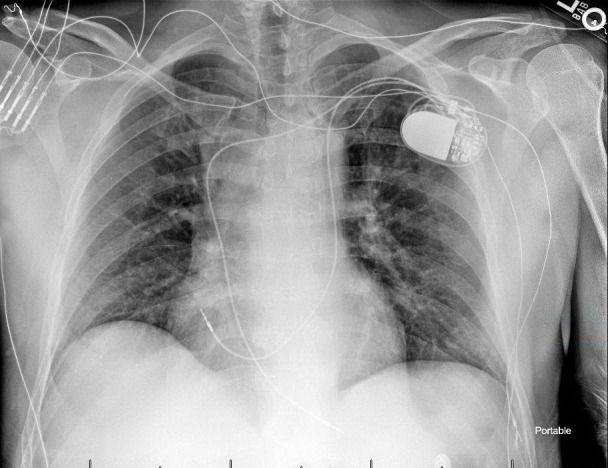
Post-implantation roentgenogram Post-procedure roentgenogram demonstrating final lead position with the right ventricular lead traversing the bridging innominate vein and the right atrial lead placed via the persistent left superior vena cava and enlarged coronary sinus.

The patient tolerated the procedure well and there were no complications. A post-procedure chest roentgenogram showed satisfactory lead positioning and no pneumothorax (Figure 1). At 16 months follow-up, he was noted to be in good health and his pacemaker was functioning normally.

## Discussion

Despite its relatively low estimated prevalence, PLSVC occurs frequently enough in high volume centers to merit consideration in cases with challenging venous access [2]. Diagnostic clues classically include caudal advancement of the guidewire at the junction of the left subclavian and internal jugular veins rather than across the midline, with confirmation by venography. Additionally, it can be detected by echocardiography, nuclear magnetic resonance, and multislice computed tomography [3]. Embryologically, this anomaly arises from failure of normal regression of the left common cardinal vein to become the ligament of Marshall. In 90% of cases of PLSVC, a right superior vena cava (RSVC) is also present, and in 30% of cases, a bridging innominate vein is observed linking the two [3]. Review of the pertinent literature reveals multiple reports of successful pacemaker, ICD, and cardiac resynchronization therapy (CRT) device placement in patients with PLSVC [2, 4].

Right ventricular (RV) lead placement via the PLSVC and coronary sinus (CS) can be technically challenging due to the acute angle created by the anomalous anatomy [5]. Exclusive utilization of the PLSVC-CS pathway may be suboptimal depending on the size of the CS, and excessive lead placement may produce hemodynamically significant effects [6]. A search for a bridging innominate vein may be advantageous for the placement of the RV lead, and it has been shown that even with negative antegrade venography, a retrograde venogram can be revealing [7]. Where the innominate vein appears stenotic, venoplasty has been described to facilitate placement of the RV lead as well [8]. Alternatively, if anomalous PLSVC-CS anatomy is known or suspected in advance, based for example on a dilated CS detected on echocardiography, a right pectoral approach may be considered if RSVC is present. This would allow for sparing of the CS in the event of future CRT upgrade, though, as noted, these patients typically have a dilated CS, which may be more amenable to multiple lead placements if needed. 

## Conclusions

The present case highlights a potential benefit of identifying and utilizing the bridging innominate vein for right ventricular (RV) lead placement. The complexity of contemporary implantable devices, and the potential need for upgrade or revision, highlight the importance of thoughtful consideration and planning vis-a-vis lead placement in the presence of PLSVC.
